# Estimation of Age-at-Death Using Cortical Bone Histomorphometry of the Rib and Femur: A Validation Study on a British Population

**DOI:** 10.3390/biology11111615

**Published:** 2022-11-04

**Authors:** Christina Karydi, Julieta Gómez García-Donas, Konstantina Tsiminikaki, Andrea Bonicelli, Konstantinos Moraitis, Elena F. Kranioti

**Affiliations:** 1 Edinburgh Unit for Forensic Anthropology, School of History Classics and Archaeology, University of Edinburgh, Edinburgh EH8 9AG, UK; 2 Department of Forensic Medicine and Toxicology, School of Medicine, National and Kapodistrian, University of Athens, 11527 Athens, Greece; 3 Center for Anatomy and Human Identification, School of Science and Engineering, University of Dundee, Scotland DD1 5EH, UK; 4 Institute of Molecular Biology and Biotechnology, University of Crete, 70013 Heraklion, Greece; 5 School of Natural Sciences, University of Central Lancashire, Preston PR1 2HE, UK; 6 Forensic Medicine Unit, Department of Forensic Sciences, Faculty of Medicine, University of Crete, 71110 Heraklion, Greece

**Keywords:** age estimation, osteons, UK, histological methods, microscopy, bone remodelling, forensic anthropology, osteology

## Abstract

**Simple Summary:**

The examination of bone microarchitecture has been considered a valuable tool for age estimation through different skeletal elements. The universal application of existing histological age-estimation techniques is, however, hindered by interpopulation variability, and therefore, validation studies are necessary to evaluate whether a specific method is adequate for accurate age estimation, or a new revised technique should be developed. This study performed a histomorphometric analysis of ribs and femora from a 19th century British population and tested the accuracy of six widely used histological age-estimation equations. The results showed that certain histomorphometric features were significantly affected by interpopulation differences. Two methods were indicated as being the most reliable for the sample under study. The research concluded that the accuracy of age-estimation methods is dependent on the demographic resemblance between the study and the reference sample.

**Abstract:**

Histomorphometry constitutes a valuable tool for age estimation. Histological interpopulation variability has been shown to affect the accuracy of age estimation techniques and therefore validation studies are required to test the accuracy of the pre-existing methodologies. The present research constitutes a validation study of widely known histological methods on the sixth rib and the femoral midshaft of a 19th century British population originating from Blackburn, England. An evaluation of the histomorphometric features of eleven ribs and five femora was performed and used to test the accuracy of selected methods. Results indicated that osteon area and circularity were the only histomorphometric variables that presented significant interpopulation variability. Cho et al.’s method for the ribs and the average value produced using Kerley and Ubelaker’s method for intact osteon and percentage of lamellar bone equations for femur were considered the only reliable markers for estimating the age on the Blackburn sample. In the case of old individuals, Goliath et al.’s method provided more satisfactory results. Overall, the present study provides evidence on the applicability of the aging histomorphometric methods on a British sample and highlights the limitations of applying histomorphometric methods developed on different reference populations than the one under investigation.

## 1. Introduction

Reconstructing an individual’s biological profile is the most important task in the field of biological and forensic anthropology. The commonly used age estimation methods for adult remains are mostly based on the macroscopic observation of degenerative changes that occur on specific articular surfaces [1,2,3,4]. These methods, however, are heavily affected by the observer’s subjective interpretation [5] and are of limited value when the skeletal elements are taphonomically altered or commingled [6]. Consequently, during the last few decades, many researchers have focused on developing new age-estimation methods based on the quantitative microscopic evaluation of cortical bone [5,7,8].

Histological age-estimation methods rely on bone remodelling, a process by which bone is constantly renewed to maintain its structural integrity and homeostasis throughout the adult life [9,10]. Vascular canals are initially formed as blood vessels are incorporated in the circumferential lamellae (non-Haversian canals) [11]. As age progresses, the number of non-Haversian canals decreases, with an increase in the number of secondary osteons [11,12]. Ultimately, osteon number reaches an asymptote, occupying the entire cortical bone, with continuous remodelling leading to the increase in osteon fragments [11]. Other parameters such as osteon area and osteon circularity have been identified to change significantly throughout individuals’ age [13,14,15,16]. Differences in cortical bone microarchitecture and bone turnover rate have been observed between and within skeletal elements [17,18,19], and as a result of pathological conditions [20] and physical activity [21,22]. Physiological differences have additionally been noticed between sexes [23,24,25] and groups of different ancestry [7,23], due to hormonal, genetic, environmental and social conditions specific to the individual and/or to the populations under study.

Kerley [12] was the first to develop an age-estimation method based on the histological evaluation of the midshaft of the femur, tibia and fibula. The femur has attracted much attention for the development of ageing methods, although the high histomorphometric variability of this skeletal element resulting from increased biomechanical forces must be taken into consideration [17,21]. For this reason, histological methods on ribs emerged as an alternative [7,26,27,28], since these bones are mostly subjected to respiratory movements occurring similarly across individuals [29].

Even though many histological methods have been developed over the years, these are often overlooked for estimating age at death due to their specific requirements, such as sample destruction and special equipment and training [30]. Other methodological issues such as sampling area and detailed parameters definition, as well as intrinsic sample characteristics must be taken into consideration [31]. Most of the published methodologies have been limited to one reference population, producing more accurate age estimates when tested on populations closely related to the original sample [32]. The performance of validation studies is therefore necessary to assess the accuracy of the existing methods, and to identify potential factors affecting these results [8]. Both weight-bearing and non-weight-bearing bones should be evaluated to better identify the sources of intra and interpopulation variability [32].

The aim of this study is to investigate the femur and rib aging histomorphometric parameters of a 19th century British population and to test the reliability of exiting histological methods when applied to a British sample. The equations selected were developed by Kerley [12]/Kerley and Ubelaker [33], Alhqvist and Damsten [34], Stout and Paine [28], Stout et al. [26], Cho et al. [7], and Goliath et al. [16] on individuals of European-American ancestry.

## 2. Materials and Methods

### 2.1. Sample

The sample under study consisted of 14 individuals recovered from St. Peter’s burial ground in Blackburn, who died between 1839 and 1857. The thin sections were retrieved from the middle third of the 6th rib (5th or 7th in case of fragmentation or unavailability of the 6th) and left or right mid-shaft of the femur. Age and sex of the individuals was known through cemetery records.

Due to taphonomic damage, the selection of specimens was limited in number and demographic profile. The rib sample (Table 1) consisted of five males, five females and one individual of unknown sex. The known age-at-death range was 1.5–69 years, with a mean and standard deviation of 35.2 ± 21.8 years. The age range for males was 29–43 years (38 ± 5.5 years) and for females 2.8–69 years (mean = 39.2, SD = 29.1 years). Two specimens (rib_7 and rib_11) were evidently pathological due to abnormal bone overgrowths but were included in the study since histological methods should be applicable to any recovered elements [33]. The femur sample (Table 1) consisted of five males with an age range of 25 to 72 years (mean = 51.8, SD = 20.2 years).

### 2.2. Sample Preparation and Data Acquisition

The thin sectioning of the sample was performed according to the methodology described by García-Donas et al. [35]. Τhe histomorphometric variables were examined using a binocular transmitted light standard research microscope (Leica DM300), equipped with 10× oculars and 4× and 10× objectives. Image capture was performed using a camera (Leica MC170HD) connected by means of a C-mount (0.55×) adapter on a transmitted light microscope (Leica DM750P) and linked to the Leica Application Suite (LAS 4.6) image acquisition software. ImageJ (1.52) software was used for metric data collection.

For the rib specimens, four histological methods were evaluated: Stout and Paine [28], Stout et al. [26], Cho et al. [7] and Goliath et al. [16]. Although the sampling locations of the above methods slightly differ, histological findings are interchangeable between different ribs [36] and sections of the same rib [19,37]. Seven histomorphometric variables were assessed as indicated by the original methods (Table 2). For the Cho et al. [7] method, only the European-American (EA) equation was tested, given the European ancestry of the sample under study.

**Figure 1:**
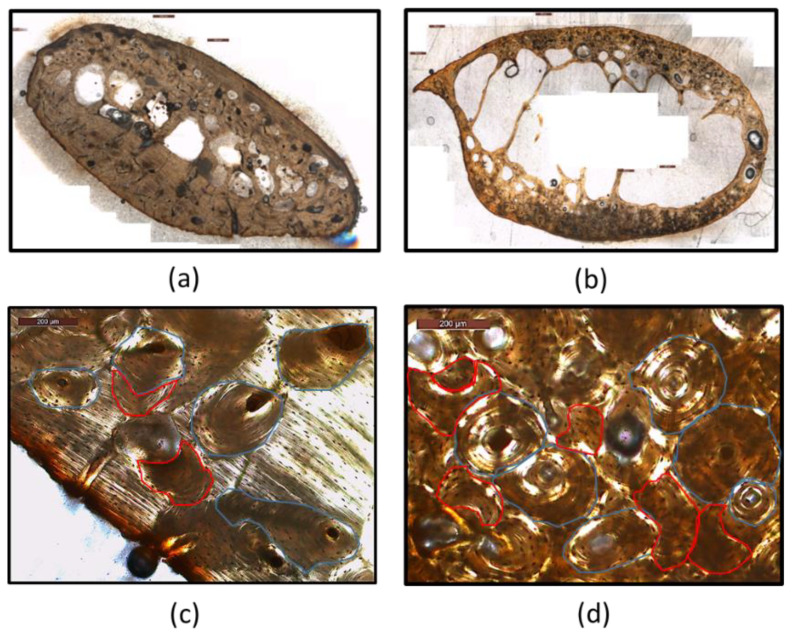
Age-related differences in the microstructure of cortical bone. Note the differences in cortical thickness and osteon density between rib 4 (1.5 years old) and rib 2 (69 years old). (**a**) Cross-section of rib 4; (**b**) cross-section of rib 2; (**c**) rib 4 and (**d**) rib 2 indicating examples of intact osteons (blue outline) and osteon fragments (red outline).

From the assessed variables, the intact and fragmented osteon number was measured using the microscope calibrated at 100× magnification. Total, trabecular and cortical area were measured digitally, instead of using the Merz counting reticule indicated by Stout and Paine [28] (Figure 1). Specifically, microphotographs of the rib cross-section were captured using the microscope camera, calibrated at 40× magnification. Using the LAS 4.6 program, the microphotographs were stitched together, reconstructing the entire cross-section. The images were then imported into the ImageJ software and the total and trabecular area were manually outlined and measured using the polygon tool selection. Cortical area was then calculated by subtracting the trabecular area from the total area.

Regarding osteon area and circularity, according to the authors’ suggestions [7,16] only structurally complete osteons with round Haversian canals should be measured. Drifting, irregular, and elongated osteons with non-circular Haversian canals were excluded from the measurements. Semi-polarized microphotographs, calibrated at 100× magnification, were captured from different areas of the cross-section. The area and shape descriptors of ImageJ were selected, and the parameters were measured using the polygon tool selection. A minimum of 25 to a maximum of 35 osteons were measured according to the original methodologies. Examples of age-related differences in bone histomorphometry are presented in Figure 1.

Three histological methods applicable on the femoral midshaft were selected for the evaluation of the femur sample, those developed by Kerley [12]/ Kerley and Ubelaker [33], Ahlqvist and Damsten [34] and Goliath et al. [16]. Given the extent of taphonomic alteration, the periosteal area could not be examined, leading to the positioning of the microscopic fields in the middle third of the cortex. As a result, a similar procedure was followed for the Kerley [12], Kerley and Ubelaker [33] and Goliath et al. [16] methods for consistency. Several images of the anterior, medial, lateral and posterior areas of the cross-section were captured at 100× magnification and stitched together. The microscopic fields were then defined by drawing a circle using ImageJ with a diameter of 1.62 mm and an area of 2.06 mm^2^, according to Kerley and Ubelaker’s [33] methodology. The femoral histological variables were examined according to the descriptions of the original studies (Table 2). Osteon area and circularity were measured on five to eight osteons in each location [16] using semi-polarised microphotographs captured at 100× magnification.

The microscopic fields analysed for the Ahlqvist and Damsten method [34] were positioned, between Kerley’s [12] and Goliath’s et al. [16] fields, in the anteromedial, anterolateral, posteromedial and posterolateral areas, following a similar procedure. The grid required for the examination of the percentage of the osteonal bone was not adjusted to the microscope as in the original methodology but was designed digitally. The grid tool option was enabled in Image J, and a 1 mm^2^ square grid comprised of 100 squares was drawn. Consulting both the images and the microscope, the number of squares half-filled with osteonal bone were recorded at each location and the average amount was given as the total percentage of osteonal bone.

### 2.3. Statistical Analysis

Intra-observer error was evaluated using the technical error of measurement (TEM) analysis. All the histomorphometric variables were re-examined for two rib specimens (18% of the sample), since at least 10% of the data should be remeasured to estimate reliability [38]. Relatively old individuals (43 years old and 69 years old) were assessed. As age increases, so does the osteon number, which in turn increases the probability for inconsistency with low intra-observer error being reported indicating high repeatability. The TEM, %TEM indices and the R coefficient of reliability were calculated [39]. A slightly different procedure was followed for the On.Ar and On.Cr variables, since these represent the mean of several measurements. The SD in this case was calculated using all the individual osteon measurements. The error of measurement was considered acceptable when the rTEM index the R coefficient were higher than 95% and 0.75, respectively [40,41]. The repeatability of the measurements is ensured when at least 95% of their variability is free of error and the R coefficient is close to 1.

The mean of the variables included in the equations, as well as the age and sex composition of the sample for each skeletal element were compared with the published values of the reference samples, using a one-sample t-test, which is accurate for small sample sizes [42]. Age-related changes in the histomorphometric values were explored by calculating Pearson’s correlation coefficient (r). Sex differences were also evaluated for the rib specimens using ANCOVA to control for the differences in the age distribution. Each statistical test was used after the sample was tested for meeting the required assumptions [43].

The reliability of each method was determined by reporting the absolute error range and by calculating the bias and inaccuracy of the age estimates according to Lovejoy et al. [44]. For the rib sample, bias, inaccuracy, and absolute error range were also calculated for different sub-categories depending on the age group and the sex. Three age groups were determined to best fit the sample: juveniles, adults <40 years old and adults >40 years old.

One-way ANOVA, ANCOVA and the Welch test were used to investigate sex differences in the accuracy of each rib method. Such an analysis was not performed for the femur sample due to the lack of female specimens. The agreement between known and estimated ages was additionally assessed using the Bland–Altman plot [45]. In each plot, the mean difference between the estimated and actual ages, the best fit line, the 95% limits of agreement and the reported errors of each methodology (95% confidence interval of SEE) were depicted. Paired-sample t-tests and the sign test were additionally employed to test for statistically significant differences between the true and estimated values. SPSS software (IBM SPSS Statistics for Windows, Version 22.0. Armonk, NY, USA: IBM Corp.) was used for the statistical analysis.

## 3. Results

### 3.1. Intra-Observer Error

The reliability of the measurements was established since relative TEM and R values fell within the expected threshold (%TEM < 5% and R > 0.75, Table 3).

### 3.2. Rib Analysis

#### 3.2.1. Histomorphometric Analysis

Summary statistics for the age composition and the histomorphometric data of the current and reference samples are presented in Table 4. The age distribution of the rib sample (mean = 35.2 years, SD = 21.8 years) was statistically lower only for the Goliath et al. value [16] [t(10) = −4.076, *p* = 0.02], but no significant differences were detected for the other methods [7,26,28]. Similar results were achieved for the OPD variable [t(10) = −3.222, *p* = 0.009]. Ct.Ar/Tt.Ar was found to be similar between the Blackburn and Cho et al.’s [7] specimens, but On.Ar and On.Cr were statistically different from the reported values for both methods (for On.Ar: t(344) = −3.795, *p* = 0.000 [7] and t(344) = 8.860, *p* = 0.000 [16]; for On.Cr: t(344) = 3.814, *p* = 0.00 [16]). The possibility of the inclusion of juveniles or pathological specimens in the sample as a cause for these statistically significant differences was explored but both variables remained statistically different from the reported results.

All histomorphometric features were significantly correlated with age, except for On.Ar which only indicated a slight decrease with age (r = −0.044). OPDI (r = 0.635), OPDF (r = 0.922), OPD (r = 0.930) and On.Cr (r = 0.775) were positively correlated with age, while Ct.Ar/Tt.Ar (r = −0.781) exhibited a negative trend. Females demonstrated lower On.Ar, On.Cr and Ct.Ar/Tt.Ar values and higher OPD values compared to males, but these differences were not statistically significant when the age factor was controlled (Table 5).

#### 3.2.2. Age Estimation Methods

**Figure 2:**
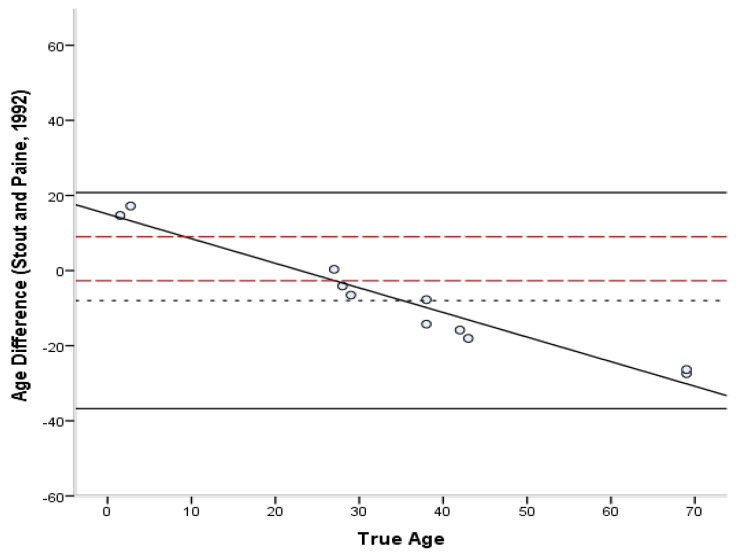
Bland–Altman plot for Stout and Paine’s [28] method illustrating the differences between known and estimated ages for the ribs against the known age. Depicted are 95% limits of agreement (black horizontal lines), mean difference/bias (black dotted line), best fit lines and 95% confidence intervals of the SEE for each method (dashed lines). R^2^ of the best fit line: 0.947.

**Figure 3:**
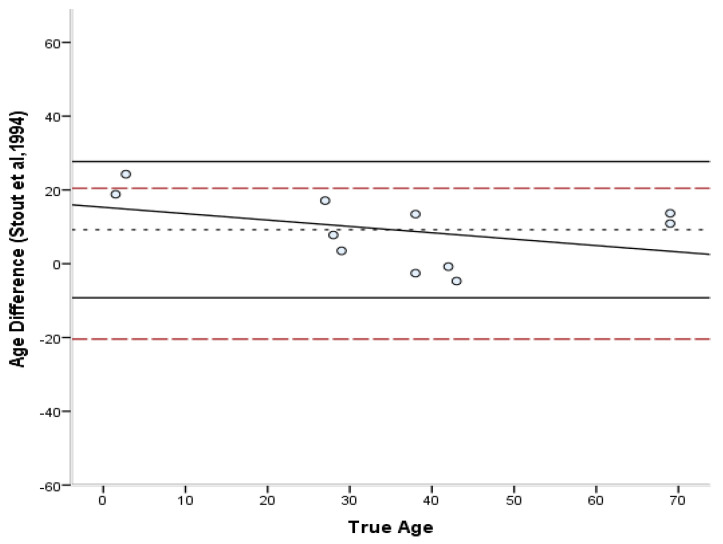
Bland–Altman plot for Stout’s et al. [26] method illustrating the differences between known and estimated ages for the ribs against the known age. Depicted are 95% limits of agreement (black horizontal lines), mean difference/bias (black dotted line), best fit lines and 95% confidence intervals of the SEE for each method (dashed lines). R^2^ of the best fit line: 0.160.

**Figure 4:**
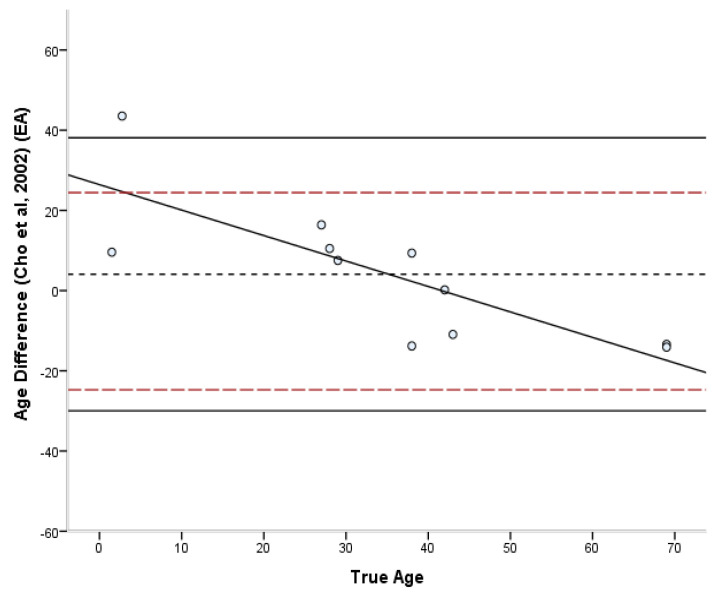
Bland–Altman plot for Cho’s et al. [7] method illustrating the differences between known and estimated ages for the ribs against the known age. Depicted are 95% limits of agreement (black horizontal lines), mean difference/bias (black dotted line), best fit lines and 95% confidence intervals of the SEE for each method (dashed lines). R^2^ of the best fit line: 0.636.

**Figure 5:**
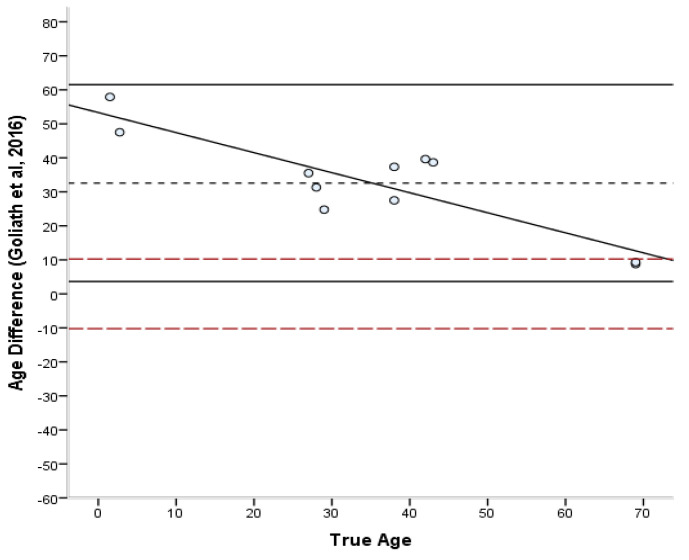
Bland–Altman plot for Goliath’s et al. [16] method illustrating the differences between known and estimated ages for the ribs against the known age. Depicted are 95% limits of agreement (black horizontal lines), mean difference/bias (black dotted line), best fit lines and 95% confidence intervals of the SEE for each method (dashed lines). R^2^ of the best fit line: 0.758.

The estimated ages according to each method are presented in Appendix A (Table A1). The bias, inaccuracy, and absolute error range of the age estimates produced by each method are summarized in Table 6 and Table 7. The absolute differences varied from 0.3 to 27.5 years for the Stout and Paine [28] method, 0.8 to 24.3 years for the Stout et al. [26] method, 0.2 to 43.5 years for the Cho et al. [7] equation, and 8.8 to 57.9 years for the Goliath et al. [16] method. For the entire sample, the lowest bias was demonstrated by the Cho et al. [7] equation (4.07 years) and the lowest inaccuracy (10.68 years) by the Stout et al. [26] method. Overall, most formulae overestimated the age of young individuals and underestimated the age of old individuals. Only the Stout et al. [26] and Goliath et al. [16] methods showed a consistent over-estimation as a general pattern, which was more pronounced in the latter. When different age groups were considered separately, adults <40 years old were more accurately estimated by the Stout and Paine method [28], while adults >40 years old by the Stout et al. [26] method. When the Bland–Altman plots (Figure 2, Figure 3, Figure 4 and Figure 5) were additionally considered, it was evident that only the Goliath et al. [16] method more accurately estimates age of the older specimens (Rib_2 and Rib_5, 69 y.o.). Statistically significant sex differences in the age estimates (Table 7) were not discovered when the age factor was controlled for, excluding for the Stout et al. [26] formula, in which males were more accurately estimated than females (*p* = 0.039).

The differences between the known and estimated ages were assessed through Bland–Altman plots [45] (Figure 2, Figure 3, Figure 4 and Figure 5). In the Stout and Paine [28] method, there was no reported SEE, and therefore the 95% confidence interval of the SEE was replaced by the error range of their sample (−2.7 to +9 years). The differences between known and estimated ages did not exceed the expected errors of the Stout et al. [26] and Cho et al. [7] equations, with the exception of a juvenile specimen, but were well above the reported error limits of the Stout and Paine [28] and Goliath et al. [16] methods. Paired-sample t-tests demonstrated that only the Stout et al. [26] and Goliath et al. [16] formulae yielded statistically different age estimates from the known age of the individual (t(10) = 3.241, *p* = 0.009 and t(10) = −7.316, *p* < 0.001, respectively).

### 3.3. Femur Analysis

#### 3.3.1. Histomorphometric Analysis

Summary statistics for the age composition and the histomorphometric data of the current and reference samples are presented in Table 8. Due to the high age of the femur sample non-Haversian canals were not found in any of the observed cross-sections. No significant differences were found in the age distribution between the Blackburn femur sample (mean = 51.8 years, SD = 20.2 years) and the reference samples. Since Kerley [12,33] and Ahlqvist and Damsten [34] did not provide any descriptive statistics, the histomorphometric variables were only compared to the values reported by Goliath and colleagues [16]. On.Ar and On.Cr of the Blackburn specimens were found statistically lower (*p* = 0.001), but there was no difference in the OPD (*p* = 0.777) between the two samples.

For the sample under study, osteon circularity was significantly increased with age (r = 0.932, *p* = 0.021), while the other variables followed a non-statistically significant trend: OPDI, OPD and percentage of osteonal bone increased with age (r = 0.925, r = 0.631, r = 0.327, respectively), while the percentage of lamellar bone and On.Ar decreased (r = −0.535 and r = −0.388, respectively). OPDF did not exhibit any pattern (r = −0.065). It must be noted, however, that one specimen (femur_33) was pathological and exhibited very thin cortical bone and large Haversian canals. Therefore, this specimen was omitted from subsequent examination and methodologies that included osteon numbers, but was retained for the rest of the analysis, since it did not produce any significant outliers for the rest of the variables.

#### 3.3.2. Age Estimation Methods

For Kerley’s [12,33] method, age was estimated using the intact osteon, fragmented osteon and percentage of lamellar bone equations. Different combinations of these equations were also tested to potentially identify the most accurate outcome. However, the equation developed on the number of fragmented osteons presented some technical issues. Even though femur_33 was omitted from this equation as mentioned above, this individual’s age was calculated by the current formula for demonstration purposes. This femur belonged to a 72-year-old individual, and although it exhibited the highest number of fragments (136), the age was highly underestimated. The number of fragments counted for each specimen and the corresponding estimated age are presented in Table 9. Originally, increasing the number of fragments, the formula yielded higher age estimates. However, after a certain cut-off point, estimated at 88 osteons, the estimated age started decreasing, leading to an estimate of 11.58 years for 136 fragments. Further increasing the number of fragments led to negative values. For this reason, and because the fragmented osteon variable did not present any correlation with age during the histomorphometric analysis presented above, this equation and its combined results, were excluded from the rest of the analysis.

The estimated ages according to each method are presented in Appendix A (Table A2). The bias, inaccuracy and absolute error range of the age estimates produced by each method are summarized in Table 10. The absolute differences between estimated and actual ages varied for Kerley’s method [33] between 6.7 and 14.8 years for the intact osteon equation, 1.6 and 43.0 years for the % lamellar bone equation and 4.5 and 14.1 years when the results of both equations were averaged. For the Ahlqvist and Damsten [34] method, absolute error was between 17.4 and 62.5 years, and for the Goliath et al. [16] method, between 2.2 and 38.9 years. When error ranges, bias and inaccuracy were considered, the average Kerley value presented the lowest inaccuracy. When each equation was considered separately, the mean difference was low for intact osteons, but the mean absolute difference increased to 11 years. The percentage of lamellar bone equation produced bias and inaccuracy of approximately 16 years, similarly to the results of Goliath et al. [16] method.

**Figure 6:**
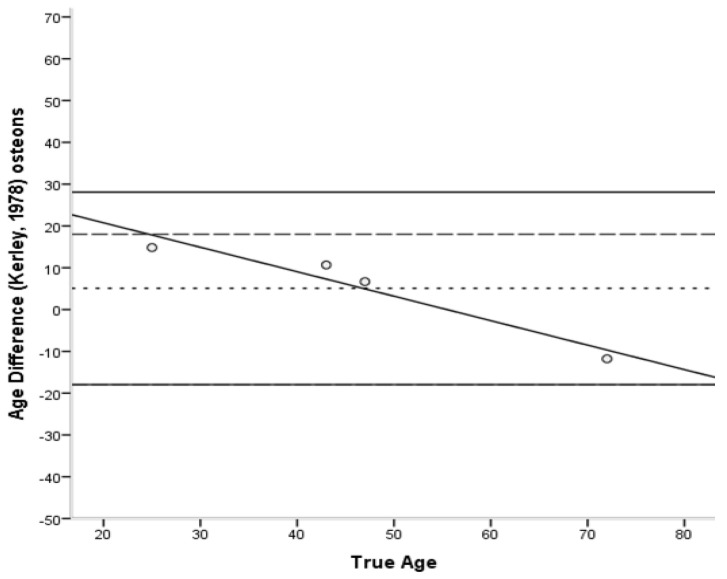
Bland–Altman plot for Kerley’s [33] intact osteon equation illustrating the differences between known and estimated ages for the femora against the known age. Depicted are 95% limits of agreement (black horizontal lines), mean difference/bias (black dotted line), best fit lines and 95% confidence intervals of the SEE for each method (dashed lines). R^2^ of the best fit line: 0.933.

**Figure 7:**
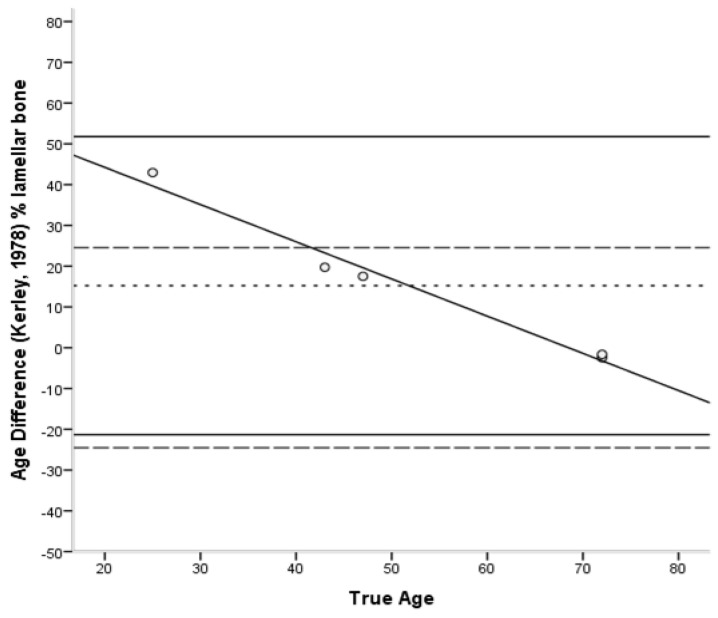
Bland–Altman plot for Kerley’s [33] % lamellar bone equation illustrating the differences between known and estimated ages for the femora against the known age. Depicted are 95% limits of agreement (black horizontal lines), mean difference/bias (black dotted line), best fit lines and 95% confidence intervals of the SEE for each method (dashed lines). R^2^ of the best fit line: 0.978.

**Figure 8:**
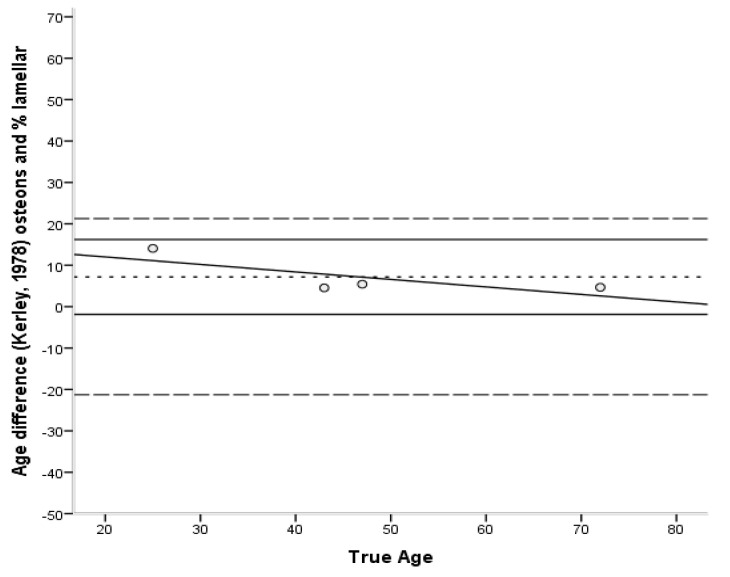
Bland–Altman plot for Kerley’s [33] intact osteon and % lamellar bone equations average value illustrating the differences between known and estimated ages for the femora against the known age. Depicted are 95% limits of agreement (black horizontal lines), mean difference/bias (black dotted line), best fit lines and 95% confidence intervals of the SEE for each method (dashed lines). R^2^ of the best fit line: 0.579.

**Figure 9:**
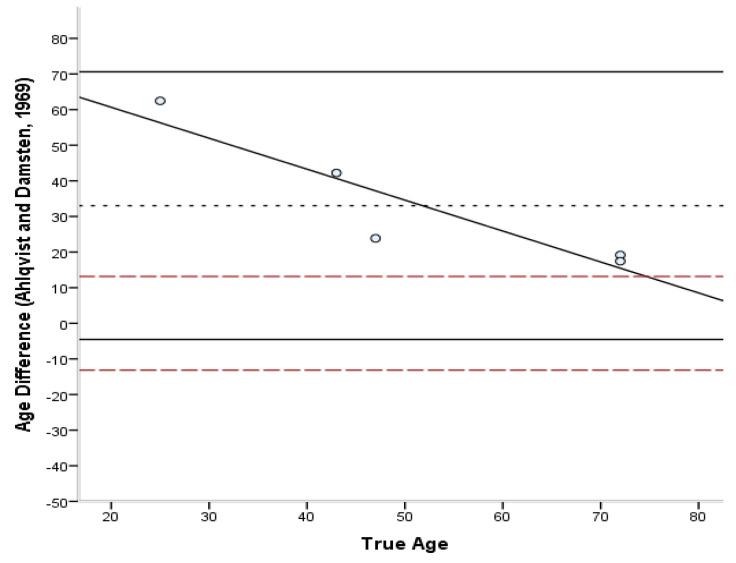
Bland–Altman plot for Alhqvist and Damsten’s [34] method illustrating the differences between known and estimated ages for the femora against the known age. Depicted are 95% limits of agreement (black horizontal lines), mean difference/bias (black dotted line), best fit lines and 95% confidence intervals of the SEE for each method (dashed lines). R^2^ of the best fit line: 0.840.

**Figure 10:**
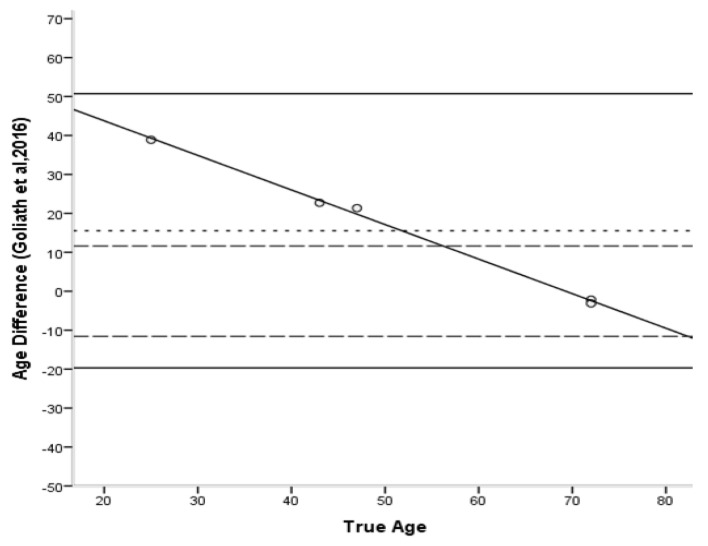
Bland–Altman plot for Goliath’s et al. [16] method illustrating the differences between known and estimated ages for the femora against the known age. Depicted are 95% limits of agreement (black horizontal lines), mean difference/bias (black dotted line), best fit lines and 95% confidence intervals of the SEE for each method (dashed lines). R^2^ of the best fit line: 0.997.

The Bland–Altman plots [45] (Figure 6, Figure 7, Figure 8, Figure 9 and Figure 10) indicated that the majority of equations overestimated the age of young individuals and underestimated the age of old individuals. Only Kerley’s [12,33] average value and the equation developed by Ahlqvist and Damsten [34] showed a consistent over-estimation as a general pattern. For the majority of the equations, older adults were more accurately estimated. Kerley’s [12,33] average value (range: 18.1 years) showed the tightest limits of agreement, followed by the intact osteon equation (46 years). Paired-sample t-tests and the sign test demonstrated that only the Ahlqvist and Damsten [34] method produced statistically higher age estimates (t(4) = −3.852, *p* = 0.018).

### 3.4. Differences in Femur and Rib Histomorphometrics

The final step of this analysis included the evaluation of the differences in the OPD, On.Ar and On.Cr variables between the two skeletal elements. OPDI and OPDF were not compared, since different counting standards were used between the two bones. As a general pattern, femur values were greater than rib values (Table 11); however, only On.Ar was statistically different between the two bones when controlling for the different age distribution between the two samples.

## 4. Discussion

Histomorphometry is a valuable method for age estimation when the integrity of skeletal remains has been compromised. Age-estimation methods are however adapted to the demographic and histomorphometric characteristics of the population they were developed on [46,47], and usually produce inaccurate estimates when applied to other populations [8,27]. The evaluation of the histological features and the demographic characteristics of the study sample should always be considered a necessary procedure in validation studies.

### 4.1. Histomorphomeric Analysis

The mean age and related OPD of the Blackburn rib and femur specimens were similar to all the reference values, except for Goliath et al. [16] method for the ribs, accounting for the high errors of the age estimates in the latter. Even though bone remodelling and consequently OPD, are affected by physiological, environmental, and socioeconomic factors [29,32,48,49,50], this study showed that osteon density values are similar between samples of similar age and between population with similar biogeographical background. All the osteon number variables showed significant correlation with age in the rib specimens but not in the femur specimens. The lack of statistical significance for the femoral OPDI variable might illustrate that the sample size might be too small to confirm the depicted relationship rather than indicate that this variable was not affected by age-related changes.

Conversely, femoral OPDF (r = −0.065) showed a slightly negative, but almost non-existent correlation with age, and consequently OPD demonstrated a lower coefficient (r = 0.631) compared to rib specimens. Ribs are subjected to higher remodelling rates in relation to the femora [51,52], producing higher numbers of osteons and osteon fragments in a given time [53]. The smaller age range of the femur sample accompanied by a lower remodelling rate could potentially obscure any expected patterns. Alternatively, it could be the sampling location and not the skeletal element that affected the correlation. Aiello and Molleson [46] indicated that the histological features of the outer third of the cortex better reflected the age-related changes compared to the mesosteal area. Correlation between age and fragmented osteon number on the ribs but not the femora has also been indicated by Crowder [32]. His sample location included the anterior periosteal area of the femur, challenging Aiello and Molleson’s [46] opinion regarding the increased reliability of the periphery of the cortex. The histological analysis of highly mechanically affected locations might therefore be avoided and histomorphometric data should instead be collected from multiple sampling locations.

Regarding the percentage of lamellar and osteonal bone, low correlation coefficients (r = −0.535 and r = 0.327, respectively) with age have been indicated before in similar British populations, and were attributed to the retainment of the bone’s youthful appearance for greater time periods, as a specific population trait [46]. However, the lack of age-related trends could potentially be attributed to the small sample size in addition to the demography limitations, resulting in sex and age bias.

Converse to osteon density, osteon size and circularity in both ribs and femora were significantly different from the reference samples regardless sample sex and age composition. Additionally, osteon area was the only variable that did not show any significant correlation with age in either ribs or femora. Pfeiffer [54] reported that in three populations, including the Spitalfields sample, which constitutes a population of similar geographic and temporal origin as the Blackburn sample, osteon area did not exhibit significant age-related patterns for any skeletal element. However, this observation was contradicted later by Crowder [32] who demonstrated a strong relationship between On.Ar and the age of the Spitalfields collection.

Population differences in the osteon size and lack of correlation with age could be attributed to external factors that overshadow the age-related patterns. It was reported that osteon sizes vary between early 18th century and more contemporary European individuals, but not between South African and European populations, suggesting that environmental factors play a larger role in explaining interpopulation variability than genetic factors [55]. In support of this statement, great osteon sizes, of similar values to the Blackburn sample, have been found on the Spitalfields collection as well [54], but not on earlier British populations [49].

Bone microarchitecture is indeed greatly affected by the imposing biomechanical forces which reversely affect osteon diameter [21,22]. This phenomenon is even greater in weight-bearing bones such as the femur. Chronic metabolic disease can also affect osteon dimensions mostly in ribs, since they are more metabolically active than the femora [56]. If population differences are not considered, the differential sampling procedure between researchers could account for this lack of agreement. The authors who incorporate this variable in their age estimation equations do not explicitly define their standards for osteon selection. Since there is a great variability in osteon sizes within the same thin section [54], different results might be produced in the absence of specific standards.

On.Cr is the only variable consistently related to age in both femur and rib. All studies indicate that osteon circularity increases with age [15,16,57]. Regardless of its strong correlation with age, the average value deviated from the literature. Physical activity and differences in biomechanical stimulation can cause intra- and interpopulation variability [21,22,58]. Imposing biomechanical forces are suggested to influence osteon circularity since more circular shapes offer greater resistance under conditions of high mechanical loading [58].

Finally, no significant sex differences in the histological features were found in this study, although females scored lower On.Ar, On.Cr and Ct.Ar/Tt.Ar values, but higher OPD values. Differences were found, however, between the two skeletal elements, with femora exhibiting significantly greater osteon sizes. Although many studies support that smaller osteons are expected to be found in bones subjected to greater strain [22], greater osteon diameter in femur specimens has been reported [16,54] and were attributed to the greater size [54] and robusticity [29,59] of this skeletal element. Indeed, Pitfield and colleagues [60] reported significantly smaller osteon sizes in humeri compared to ribs in three juvenile populations, but greater osteon size in humeri of older children compared to younger children as a result of size differences. It seems therefore that the size effect of the two skeletal elements of the Blackburn sample overpowers the expected difference due to mechanical stress, resulting in bigger osteons in the femur specimens.

### 4.2. Histological Methods

The main intention of this study was to examine whether the tested age estimation methods accurately estimated the age of a 19th century British population based on the histomorphometric evaluation of the ribs of European-American individuals. Even though the sample size of the current study was small, these preliminary results are valuable in indicating the potentiality of each method, research strategies and possible problem areas.

#### 4.2.1. Rib Methods

Overall, most of the methods resulted in a consistent over-estimation of the age of juveniles and under-estimation of older individuals. The distinct microarchitecture of juvenile bones leads to high inaccuracies when adult age equations are used, and separate standards and variables must be set [61]. Conversely, the increased inaccuracy presented in older ages is consistent with the OPD asymptote observed around 60 years of age for the ribs [32]. Goliath’s et al. [16] innovation of incorporating the osteon circularity in the age- estimated formulae and omitting the OPD variable explains why this method is indicated as being the most accurate for old individuals [37,62]. However, this method demonstrated the highest errors and was proven to be inadequate for younger age groups. For this reason, it should not be applied to unknown recovered bone fragments on its own, but it could constitute a complementary procedure when other methods indicate an adult over 50 years of age.

The Stout and Paine [28] formula resulted in the under- estimated of the true age for all age groups and the sample as a whole, excluding the sub-adults. This trend was indicated by other validation studies [37,62,63] and could possibly be attributed: (1) to the logarithmic nature of the estimation formula: transforming logarithmic data to arithmetic units leads to biased estimates, underestimating the true value [64] and (2) to differences in the age and sex composition between the samples. Indeed, their reference sample had noticeably fewer females than males and a lower age variation (SD: 12.9 vs. 21.8). However, since the OPD of the current sample is similar to the OPD of their sample (18.112 ± 5.640 vs. 18.03 ± 7.19), the first reason seems to apply more firmly. Although relatively low values of bias and inaccuracy were yielded for the pooled data, the error of estimates was well above the reported one.

The Stout et al. [26] method presents controversial results. It demonstrated the tightest limits of agreement, the lowest rates of inaccuracy, and errors comparable to the published results. Additionally, sex bias was found, and the estimated ages were shown to be statistically higher than the true ages. However, the efficiency of the paired sample t-test is influenced by extreme values [65], and thus outliers could have an extreme impact on its results. Nevertheless, the reported differences could be attributed to the sample location, since Stout et al. [26] studied the sternal end of the 4th rib instead of the midshaft of the 6th rib that was used in this study. Indeed, Crowder and Rosella [36] observed lower osteon numbers in 3rd–8th ribs compared to the 6th rib. In agreement with this, higher but non-statistically significant OPD was indicated for the current sample in relation to the 4th rib [26]. This difference was escalated due to the squared regression, leading to statistically different age estimates. As a result, this study was not conclusive regarding the reliability of Stout et al. [26] method for the Blackburn population. Further testing on a larger sample is necessary.

The Cho et al. [7] method yielded the best results, with a bias of 4.07 years and an inaccuracy of 13.57 years. The differences between known and estimated ages all fell within the 95% confidence interval of the error of estimate reported by the authors, except for a sub-adult specimen. Since the mean of the estimated ages was found to be identical with the mean of the known ages, the reliability of this method for the age-estimation of the Blackburn sample was confirmed. This method was also considered the most suitable for the age estimation of the Spitalfields collection [32] confirming the repeatability of the results between similar populations.

Statistical comparisons between healthy and pathological specimens were not possible due to the small sample size. In any case, since the majority of the pathological conditions affect the remodelling rate [20] and consequently the number of intact and fragmented osteons, formulae developed on other variables, such as On.Cr, could be more reliable for the age-estimation of pathological specimens.

Sex differences were observed in the Stout et al. [26] method, with the males being more accurately estimated. The same pattern has been indicated in other studies [27], following similar sex differences in the histomorphometric variables. In this case, such differences were not noted; however, the slightly higher OPD in females (Table 5) was transformed in a high difference of age estimates due to the nature of Stout et al. [26] regression formula.

#### 4.2.2. Femur Methods

For Kerley’s method [33] the equations developed on the intact osteon number and the percentage of lamellar bone as well as their average value, were tested on the Blackburn population. In each case, estimated ages were not statistically different from true ages and the limits of agreement were slightly broader towards the positive side. Averaging the estimated ages from the two formulae proved to be the most reliable for the Blackburn population, since it exhibited the lowest inaccuracy and the tightest limits of agreement. It is not confirmed however, if this method would also work well on the periosteal area, due to its slower remodelling rate [23,66].

Reliable estimates were also obtained by the intact osteon equation. The percentage of lamellar bone formula showed an overall positive bias which is probably attributed to the highest remodelling rate observed in the middle cortical area of the femur [23,66]. The higher accuracy in older ages (72 years old) is explained by the OPD asymptote that occurs at around 70 years for the femur [32] neutralizing the high remodelling effect. The equation developed on the fragmented osteon number was proven to be inadequate, since both Aiello and Molleson [46] and this study showed that this formula analogically yields higher age estimates increasing the number of fragmented osteons in the microscopic fields, until about 90 fragments, after which further increase in their number yields to lower age estimates, even producing negative values when osteon fragment concentration is too high. If population differences are set aside, the higher remodelling rate of the middle-cortex [66] possibly accounts for the higher number of fragments and the reverse effect of Kerley’s formula.

Ahlqvist and Damsten’s [34] method demonstrated the lowest accuracy, and statically higher age estimates. All the differences between known and estimated ages fell outside the confidence interval provided by the authors, with the older individuals exhibiting the lowest errors.

The age estimates generated by the Goliath et al. [16] equation were not significantly different from the true values, although the error of the estimation was high, especially for the 25 year old individual. It must be noted however that is method accurately estimated the ages of the old individuals (72 years old), confirming the reliability of the method in older specimens.

### 4.3. Ribs or Femora?

Comparing the results of the Cho et al. [7] and Kerley [12,33] methods, it is apparent that greater accuracy is yielded by the latter. However, the lowest errors could be the result of the smaller sample size and the lack of juvenile and pathological specimens. Furthermore, it is uncertain whether this method would perform well on the less remodelled periosteal area. Differences in bone remodelling have been indicated even within the same cross-section, between sub-periosteal and endosteal areas [66,67], and therefore interstudy variation could be expected. This phenomenon is especially problematic in archaeological specimens whose sub-periosteal regions are often affected by diagenetic processes, hindering the repeatability of studies performed in the exterior parts of the cortex. An optimal solution would be the evaluation of the mesosteal area universally in all histological studies, since it constitutes a location that is rarely affected by mechanical stress and diagenetic or resorption processes. The use of osteon counts rather than osteon densities is another drawback of Kerley’s [12,33] method, since one or more of the four locations may be unavailable or affected by diagenetic changes in archaeological specimens. Relative indices are more useful in the archaeological field. Finally, Kerley’s definition of intact osteons presents technical difficulties since it is not always possible to assess if more than 20% is obscured by adjacent features.

Generally, it seems that rib methods should be more accurate and demonstrate less interobserver variability than femur methods. Sampling location is not the major cause of errors in rib methodologies since the entire cross-section can be evaluated. Additionally, there should not be any differences between ribs 3rd–8th [36] or between different locations across the rib shaft [37], but further testing should ideally be performed to confirm these results. Conversely, the evaluation of the complete femoral cross-section is impossible, and the more subjective variables a researcher adds, the greater the probability for inconsistency. It is not negligent however that ribs also exhibit certain disadvantages. They reach the OPD asymptote earlier than the femora (50 vs. 70 years) [32,47] due to their higher remodelling rate, and they are highly influenced by metabolic disease [29].

## 5. Conclusions

The current research constitutes a validation study of four methods developed on ribs and three methods developed on femora on a 19th century British population originating from Blackburn, England. This research demonstrated that the accuracy of each method is dependent on differences in the demographics between the reference and the study sample, followed by similar differences in the OPD value. Osteon area and circularity values were found to be highly population-dependent, in contrast to osteon population density and relative cortical area.

Our preliminary results indicated that the method developed by Cho et al. [7] and the mean value of the intact osteon number and % lamellar bone equations developed by Kerley and Ubelaker [33] were the most accurate age predictors of the whole Blackburn sample. In the case of juveniles, however, both methods highly overestimated their true ages. Conversely, the method developed by Goliath and colleagues [16] was promising for estimating age of older individuals; therefore, it is proposed as a complementary tool when other age-estimation methods indicate an individual around the age of the OPD asymptote. Statistical comparisons between healthy and pathological individuals were not possible, even though no significant outliers were produced by the pathological specimens. Finally, rib methods should be preferred over femur methods. The latter demonstrate excessive variability arising from different sampling locations and imposed biomechanical forces. Confirmation of these results in a larger sample is however necessary.

## Figures and Tables

**Table 1:** Demographics of the Blackburn sample.

Sample No	Sex	Age
Rib_4	-	1.5 y
Rib_39	F	2.8 y
Rib_16	F	27 y
Rib_18	F	28 y
Rib_20	M	29 y
Rib_6	M	38 y
Rib_7	M	38 y
Rib_11	M	42 y
Rib_8 ^1^	M	43 y
Rib_2	F	69 y
Rib_5	F	69 y
Femur_29	M	25 y
Femur_24 ^1^	M	43 y
Femur_34	M	47 y
Femur_26	M	72 y
Femur_33	M	72 y

^1^ These specimens belong to the same individual.

**Table 2:** Differences in the definition of the histomorphometric variables between rib and femur methods.

Histomorphometric Variable	Definition for Rib Methods	Definition for Femur Methods
Cortical area	the total area of the cortex in mm^2^	the total area of the cortex in mm^2^
Intact osteon number (N.On)	-	the total number of osteons that have an intact Haversian canal and can be recognized over 80% of their area, counted in all four fields [12,33]
Fragmented osteon number (N.On.Fg)	-	the number of remodelled osteons counted in all four fields [12,33]
Non-Haversian Canals	-	the number of primary vascular canals counted in all four fields [12,33]
The percentage of circumferential lamellar bone (% lamellar bone)	-	the amount of un-remodelled lamellar bone divided by the total field area, averaged for all four fields [12,33]
Intact osteon density (OPDI):	the number of osteons whose canals’ perimeter shows less than 10% of bone resorption per mm^2^ of cortical area [7,16,26,28]	intact osteon number (N.On) [12,33] per mm^2^ of cortical area [16]
Fragmentary osteon density (OPDF)	the number of remodelled osteons whose canals’ perimeter shows more than 10% of bone resorption, per mm^2^ of cortical area. Fragments without visible canal were also counted [7,16,26,28]	fragmented osteon number (N.On.F) [12,33] per mm^2^ of cortical area [16]
Osteon population density (OPD):	the sum of OPDI and OPDF [7,16,26,28]	the sum of OPDI and OPDF [16]
Osteon Area (On.Ar)	the area in mm^2^ occupied by a structurally complete osteon, including the Haversian canal [7,16]	the area in mm^2^ occupied by a structurally complete osteon, including the Haversian canal [16]
Relative Cortical Area (Ct.Ar/Tt.Ar)	the cortical area divided by the total area of the cross-section [7]	-
Osteon Circularity (On.Cr)	the degree of similarity of the osteon shape to a true circle defined by the index: (4 π(area/perimeter^2^)) [16]	the degree of similarity of the osteon shape to a true circle defined by the index: (4 π(area/perimeter^2^)) [16]
The percentage of osteonal bone (% osteonal bone)	-	the area occupied by osteons and osteon fragments in relation to the total evaluated area [34]

**Table 3:** Estimation of intra-observer error through the TEM analysis.

Variable	TEM	%TEM	R
On.N	5.59	2.64	0.89
On.N.Fg	8.02	2.28	0.97
Tt.Ar	0.21	0.28	1.00
Es.Ar	0.14	0.28	1.00
Ct.Ar	0.18	0.70	0.99
OPDI	0.19	2.31	0.96
OPDF	0.30	2.14	0.99
OPD	0.21	0.95	0.99
On.Ar	0.002	4.08	0.99
On.Cr	0.01	0.60	0.97
Ct.Ar/Tt.Ar	0.002	0.54	0.99

**Table 4:** Descriptive statistics for the age and the histomorphometric variables of the current and reference rib samples.

Variable		Stout and Paine [28]	Stout et al. [26]	Cho et al. [7]	Goliath et al. [16]	Current Sample
Age	Mean	28.600	39.200	37.824	62.960	35.200
	SD	12.900	19.090	2.413	10.215	21.800
	Min	13.000	11.000	17.000	39.000	1.500
	Max	62.000	88.000	82.000	82.000	69.000
OPD	Mean	18.030	16.010	20.071	23.590	18.110
	SD	7.180	SEM: 0.750	0.975	5.930	5.640
	Min	-	-	-	13.440	8.660
	Max	-	-	-	42.330	27.720
On.Ar.	Mean	-	-	0.039	0.024	0.035
	SD	-	-	0.001	0.009	0.220
	Min	-	-	-	0.012	0.005
	Max	-	-	-	0.043	0.155
Ct.Ar/Tt.Ar	Mean	-	-	0.343	-	0.430
	SD	-	-	0.023	-	0.139
	Min	-	-	-	-	0.215
	Max	-	-	-	-	0.750
On.Cr	Mean	-	-	-	0.905	0.915
	SD	-	-	-	0.014	0.463
	Min	-	-	-	0.864	0.636
	Max	-	-	-	0.924	0.981

**Table 5:** Descriptive statistics for the histomorphometric variables of the rib sample divided by sex.

	Males		Females		Pooled Data	
Variable	Mean	SD	Mean	SD	Mean	SD
OPD	17.516	2.228	20.598	6.640	18.112	5.640
On.Ar	0.036	0.008	0.029	0.006	0.035	0.220
On.Cr	0.920	0.020	0.910	0.020	0.915	0.463
Ct.Ar/Tt.Ar	0.409	0.065	0.388	0.123	0.430	0.139

**Table 6:** Bias, inaccuracy, and absolute error range for the age estimates of the rib sample divided by age group.

	Stout and Paine [28]			Stout et al. [26]			Cho et al. [7]			Goliath et al. [16]		
Group	Bias	Inac	Rang	Bias	Inac	Rang	Bias	Inac	Rang	Bias	Inac	Rang
Juvenile	15.9	15.9	14.7–17.2	21.5	21.5	18.8–24.3	26.6	26.6	9.6–43.5	52.7	52.7	47.5–57.9
<40	−6.5	6.6	0.3–4.3	7.8	8.9	2.6–17.1	6.0	11.5	7.5–16.4	31.3	31.3	24.8–37.3
>40	−21.9	21.9	15.8–27.5	4.8	7.5	0.8–13.7	−9.6	9.7	0.2–14.1	24.1	24.1	8.8–39.6
Total	−8.0	13.9	0.3–27.5	9.2	10.7	0.8–4.3	4.1	13.6	0.2–43.5	32.6	32.6	8.8–57.9

Inac: inaccuracy; Rang = range.

**Table 7:** Bias, inaccuracy and absolute error range for the age estimates of the rib sample divided by sex.

	Stout and Paine [28]			Stout et al. [26]			Cho et al. [7]			Goliath et al. [16]		
Group	Bias	Inac	Rang	Bias	Inac	Rang	Bias	Inac	Rang	Bias	Inac	Rang
Males	−12.5	12.5	6.5–18	1.8	4.0	0.8–13.4	−1.5	8.4	0.8–13.4	33.6	33.6	24.8–39.6
Females	−8.1	15.1	0.3–27.5	14.7	14.7	7.8–24.3	8.6	19.6	10.5–43.5	26.5	26.5	8.8–47.5
Total	−8.0	13.9	0.3–27.5	9.2	10.7	0.8–4.3	4.1	13.6	0.2–43.5	32.6	32.6	8.8–57.9

Inac: inaccuracy; Rang = range.

**Table 8:** Descriptive statistics for the age and the histomorphometric variables of the current and reference femur samples.

Variable		Kerley [12,33]	Ahlqvist and Damsten [34]	Goliath et al. [16]	Current Sample
Age	Mean	41.600	55.000	62.960	51.800
	SD	-	-	10.210	20.000
	Min	0.000	-	39.000	25.000
	Max	95.000	-	82.000	72.000
OPD	Mean	-		24.320	23.880
	SD	-	-	5.550	2.150
	Min	-	-	15.660	21.480
	Max	-	-	41.100	25.970
On.Ar.	Mean	-	-	0.034	0.047
	SD	-	-	0.013	0.027
	Min	-	-	0.014	0.006
	Max	-	-	0.065	0.140
On.Cr	Mean	-	-	0.906	0.933
	SD	-	-	0.022	0.029
	Min	-	-	0.844	0.815
	Max	-	-	0.927	0.978

**Table 9:** Number of fragmented osteons and estimated ages by Kerley’s equation.

Femur Code	Age	On.N.Fg	Age Estimation
29	25	83	78.8
24	43	64	68.1
34	47	97	77.7
26	72	78	77.2
33	72	136	11.6

**Table 10:** Bias, inaccuracy and error range of the age estimates generated by the femur methods.

Method	Bias	Inaccur.	Range
Kerley Osteons [33]	5.1	11.0	6.7–14.8
Kerley % lamellar [33]	15.2	16.9	1.6–42.9
Kerley Osteons + % lamellar [33]	7.2	7.2	4.5–14.1
Ahlqvist and Damsten [34]	33.0	33.0	17.4–62.5
Goliath et al. [16]	15.5	17.6	2.2–38.9

**Table 11:** Blackburn sample descriptive statistics for the histomorphometric variables depending on the skeletal element.

	Bone	N	Mean	SD	Std. Error Mean
**OPD**	femur	5	23.884	2.156	0.964
	rib	11	18.112	5.640	1.700
**On.Ar**	femur	5	0.047	0.007	0.003
	rib	11	0.035	0.009	0.0028
**On.Cr**	femur	5	0.933	0.006	0.0029
	rib	11	0.913	0.0195	0.0059

## Data Availability

The data generated and/or analysed during this study are available upon request.

## Appendix A. Results of the Age-Estimation Methods for the Rib and Femur Sample

**Table A1:** The known and estimated age for each rib specimen by the age-estimation methods.

Rib Code	Real Age	Stout and Paine [28] Estimated Age	Stout et al. [26] Estimated Age	Cho et al. [7] Estimated Age	Goliath et al. [16] Estimated Age
4	1.5	16.2	20.3	11.1	59.4
39	2.8	19.9	27.0	46.3	50.3
16	27.0	27.3	44.1	43.4	62.5
18	28.0	23.9	35.8	38.5	59.3
20	29.0	22.5	32.5	36.5	53.8
6	38.0	23.7	35.4	24.2	75.3
7	38.0	30.3	51.4	47.4	65.5
11	42.0	26.2	41.2	42.2	81.6
8	43.0	25.0	38.3	32.1	81.7
2	69.0	41.5	79.9	55.6	77.8
5	69.0	42.7	82.7	54.9	78.3

**Table A2:** The known and estimated age for each femur specimen by the age-estimation methods.

Femur Code	Real Age	Kerley Osteon eq. [33] Estimated Age	Kerley %lamellar eq. [33] Estimated Age	Kerley Average Value [33] Estimated Age	Ahlqvist and Damsten [34] Estimated Age	Goliath et al. [16] Estimated Age
29	25.0	39.8	68.0	53.9	87.5	63.9
24	43.0	53.7	62.7	58.2	85.2	65.7
34	47.0	53.7	64.5	59.1	70.9	68.4
26	72.0	60.2	69.6	64.9	91.2	69.8
33	72.0	30.6	70.4	50.5	89.4	68.9
